# Advances in phytochemical-derived nanotherapeutics for multimodal eradication of multidrug-resistant *Staphylococcus aureus*

**DOI:** 10.3389/fphar.2026.1866492

**Published:** 2026-06-10

**Authors:** Neha Gupta, Soham Bhattacharya, Ludovica Lela, Adrish Dutta, Immacolata Faraone, Eloy Fernández-Cusimamani, Satyajit D. Sarker, Luigi Milella, Lutfun Nahar, Olga Leuner

**Affiliations:** 1 Department of Crop Sciences and Agroforestry, Faculty of Tropical AgriSciences, Czech University of Life Sciences Prague, Prague, Czechia; 2 Department of Economic Theories, Faculty of Economics and Management, Czech University of Life Sciences Prague, Prague, Czechia; 3 Department of Agroecology and Crop Production, Faculty of Agrobiology, Food and Natural Resources, Czech University of Life Sciences Prague, Prague, Czechia; 4 Department of Health Sciences, University of Basilicata, Potenza, Italy; 5 Centre for Natural Products Discovery, School of Pharmacy and Biomolecular Sciences, Liverpool John Moores University, Liverpool, United Kingdom; 6 Laboratory of Growth Regulators, Palacký University and Institute of Experimental Botany, The Czech Academy of Sciences, Olomouc, Czechia

**Keywords:** antimicrobial resistance, multidrug-resistant *Staphylococcus aureus*, next-generation antimicrobial therapies, pharmacokinetics, phytochemical-based nanoparticles, phytometabolites

## Abstract

The past decade, particularly the post-pandemic period, has intensified the challenge of managing bacterial infections. Multidrug-resistant *Staphylococcus aureus* (MDR-SA) has emerged as a dominant cause of hospital-acquired infections, creating a sustained public health emergency and underscoring the need for alternative antimicrobial strategies. Plant-derived secondary metabolites have gained attention as promising antibacterial agents; however, their therapeutic potential is constrained by poor solubility, limited target selectivity, low drug-loading capacity, rapid metabolism, and reduced systemic bioavailability. Nanoparticle carriers provide a corrective platform by improving physicochemical stability, enhancing solubility, enabling controlled release, and strengthening pharmacokinetic and pharmacodynamic behavior. Phytochemical-based nanoparticles (phyto-NPs) form a multitarget antibacterial architecture capable of weakening bacterial defense networks through efflux pump interference, disruption of metal-ion homeostasis, and alteration of membrane permeability. These systems also induce reactive oxygen species, leading to DNA damage, protein denaturation, mitochondrial impairment, and peptidoglycan disruption. In parallel, phyto-NPs inhibit biofilm formation and quorum-sensing pathways, reducing virulence and limiting dissemination. Their ability to penetrate the extracellular matrix enhances antibiotic access and restores susceptibility in resistant strains. Recent investigations demonstrate strong activity of phyto-NPs both as independent therapeutics and as synergistic partners to conventional antibiotics. Microenvironment-responsive release, intracellular targeting, and improved delivery efficiency further strengthen their translational relevance. Curcumin-loaded nanosystems disrupt MRSA membranes and impair biofilm formation, while quercetin-loaded liposomes penetrate *S. aureus* biofilms more effectively than free quercetin. These examples illustrate the capacity of nanoscale engineering to overcome the pharmacological constraints of phytochemicals. This review examines recent advances in phyto-NP strategies targeting MDR-SA, with emphasis on phytochemical selection, nanoscale design principles, and the multifunctional antibacterial mechanisms underpinning next-generation antimicrobial development.

## 1 Introduction

The global rise of multidrug-resistant bacterial infections has created a sustained threat to healthcare systems and reduced the effectiveness of frontline antibiotics. Multidrug-resistant *Staphylococcus aureus* (MDR-SA) has emerged as a major contributor to this burden due to its ability to accumulate resistance determinants and cause severe infections ranging from pneumonia to bacteraemia (Sultan et al., 2018; Murray et al., 2022; Abebe and Birhanu, 2023). Increasing resistance to methicillin, vancomycin, tetracycline, and linezolid has further complicated clinical management and intensified the need for alternative therapeutic strategies (Jones et al., 2007; Lee et al., 2014). Additional resistance to fluoroquinolones, macrolides, aminoglycosides, and glycopeptides has reduced the effectiveness of conventional antibiotics and increased the burden of difficult-to-treat infections (Khameneh et al., 2019; Makarewicz et al., 2021; Díaz-Puertas et al., 2023).

The clinical prominence of MRSA reflects its capacity to combine extensive resistance with high virulence potential. Disease manifestations range from superficial skin infections to invasive conditions such as osteomyelitis, endocarditis, and necrotizing pneumonia, contributing to significant morbidity and mortality across healthcare settings (Abebe and Birhanu, 2023; Murray et al., 2022). Additional phenotypes, including vancomycin-resistant *S. aureus* (VRSA) and linezolid-resistant strains, further restrict therapeutic options and increase the complexity of treatment decisions (Jones et al., 2007; Lee et al., 2014; Sultan et al., 2018).

The success of *S. aureus* as a resistant pathogen stems from a diverse repertoire of molecular defense mechanisms. mecA-mediated β-lactam resistance, enzymatic detoxification pathways, and activation of multidrug efflux pumps such as NorA and MepA collectively strengthen bacterial survival under antibiotic pressure (Vestergaard et al., 2019; Abebe and Birhanu, 2023). Additional mechanisms include structural modification of cell-wall components, regulation of intracellular metal homeostasis, and induction of the SOS stress response, each contributing to enhanced adaptability and persistence (Vestergaard et al., 2019; Zhao et al., 2020; Douglas, 2022). Biofilm formation provides further protection by limiting antibiotic penetration and enabling long-term colonization on host tissues and medical devices (Monte et al., 2014; Yadav et al., 2015; Bogdanova et al., 2018).

Plant-derived phytochemicals have gained increasing attention as antibacterial agents capable of countering MDR-SA through structurally diverse and mechanistically distinct pathways. Major classes such as alkaloids, polyphenols, terpenoids, coumarins, and cannabinoids demonstrate activity against resistant strains through membrane disruption, efflux-pump inhibition, toxin suppression, and interference with quorum-sensing networks (Appendino et al., 2008; Miklasińska-Majdanik et al., 2018; Khameneh et al., 2021). Representative compounds including berberine, quercetin, curcumin, caffeic acid, and sabinene exhibit inhibitory effects across multiple MDR-SA lineages, highlighting the therapeutic potential of phytochemical scaffolds (Chung et al., 2014; Hua et al., 2018; Sinsinwar and Vadivel, 2020). Their ability to act on multiple bacterial systems reduces the likelihood of rapid resistance development and broadens their therapeutic relevance.

Despite their mechanistic diversity, phytochemicals face significant translational barriers. Poor aqueous solubility, rapid metabolic degradation, and low systemic bioavailability limit therapeutic performance and restrict clinical applicability (Huang et al., 2019; Makarewicz et al., 2021; Paul-Chima et al., 2024). Variability in plant sources and extraction methods introduces challenges in standardization and reproducibility, while high concentrations required for antibacterial activity raise concerns regarding toxicity and off-target effects (Khameneh et al., 2019; Kongkham et al., 2020; Patil et al., 2023).

Nanotechnology-based delivery systems provide a rational framework for overcoming these limitations by encapsulating phytochemicals within engineered nanocarriers. Improved solubility, enhanced stability, controlled release, and targeted delivery have been demonstrated across liposomes, polymeric nanoparticles, metallic nanoparticles, and solid-lipid nanoparticles (Jeetah et al., 2014; Naseri et al., 2015; Cano et al., 2020). Stimuli-responsive nanocarriers, including pH-sensitive and enzyme-responsive systems, enable selective release within infected microenvironments and improve clearance of intracellular MRSA (Mohammed et al., 2023; Zou et al., 2023; Ali Khan et al., 2024). Preclinical studies, including curcumin-loaded nanoparticles and quercetin-loaded liposomes, demonstrate enhanced membrane disruption, improved biofilm penetration, and superior bactericidal activity compared with free compounds (Cui et al., 2016; Kumar et al., 2022; Zhang et al., 2023). This review integrates phytochemical classes, nanocarrier design considerations, and antibacterial mechanisms to frame how phyto-nanotherapeutic strategies can address persistent resistance challenges in MDR-SA infections.

## 2 Phytochemicals: Promising antibacterial agents against MDR *S. aureus*

Phytochemicals have emerged as promising antibacterial agents in response to the growing limitations of conventional antibiotics against multidrug-resistant bacterial pathogens. Their structural diversity and capacity to act on multiple bacterial targets provide mechanistic advantages over single-target antibiotics, particularly in the context of MDR-SA. Unlike traditional antimicrobials, phytochemicals interfere with bacterial physiology through a broad range of molecular mechanisms, thereby reducing susceptibility to rapid resistance development (Khameneh et al., 2021).

The antibacterial activities of phytochemicals encompass disruption of bacterial cell walls and membranes, leading to increased permeability and leakage of intracellular contents. Several compounds impair protein synthesis, interfere with nucleic acid replication, induce DNA damage, and promote oxidative stress through reactive oxygen species generation. Phytochemicals also suppress quorum-sensing and virulence-associated signaling pathways, thereby attenuating toxin production, adhesion, and biofilm formation in MDR-SA strains (Khameneh et al., 2016; Fazly Bazzaz et al., 2018; Fatemi et al., 2020).

A defining feature of phytochemicals is their complex chemical architecture, which limits bacterial enzymatic degradation and reduces the efficiency of resistance acquisition. Many phytochemicals retain activity against resistant strains by bypassing classical resistance pathways, including altered penicillin-binding proteins and target-modification mechanisms. In addition, several phytochemicals function as resistance-modifying agents by inhibiting efflux pumps and restoring bacterial susceptibility to co-administered antibiotics (Miklasińska-Majdanik et al., 2018; Khameneh et al., 2021).

Beyond antibacterial efficacy, phytochemicals have demonstrated synergistic interactions with conventional antibiotics, enhancing bactericidal effects and reducing required antibiotic dosages. These interactions are particularly relevant in MDR-SA infections, where efflux-pump inhibition, membrane destabilization, and biofilm disruption collectively improve drug penetration and intracellular accumulation (Khameneh et al., 2019; Kongkham et al., 2020). Certain phytochemicals, including capsaicin, codeine, colchicine, and paclitaxel, have received approval from the U.S. Food and Drug Administration for various therapeutic applications, underscoring their clinical relevance (Kongkham et al., 2020).

Despite these advantages, the clinical translation of phytochemicals remains constrained by pharmacokinetic limitations. Poor aqueous solubility, chemical instability, rapid metabolic degradation, and limited bioavailability compromise systemic exposure and therapeutic consistency. Variability in plant sources, extraction procedures, and compound composition further complicates standardization and reproducibility across studies (Huang et al., 2019; Makarewicz et al., 2021; Paul-Chima et al., 2024). High concentrations required for antibacterial activity raise concerns regarding toxicity and off-target effects, reinforcing the need for improved delivery strategies (Khameneh et al., 2019; Kongkham et al., 2020; Patil et al., 2023).

Advances in high-throughput screening, analytical chemistry, and phytochemical classification have accelerated the identification of bioactive compounds and clarified their structure-activity relationships. Structural classification into alkaloids, phenolic compounds, terpenoids, coumarins, and cannabinoids provides a mechanistic framework for understanding antibacterial activity and guiding rational therapeutic development. Each phytochemical class exhibits distinct physicochemical properties and antibacterial mechanisms relevant to MDR-SA infections, as outlined in the following subsections and accompanying structural representation.

### 2.1 Alkaloids

Alkaloids are heterocyclic nitrogen-containing compounds with structurally diverse frameworks and significant antimicrobial activity. Their ability to act as hydrogen-bond donors or acceptors contributes to strong target-ligand interactions and underpins their broad antibacterial potential against MDR-SA (Kittakoop et al., 2014; Kumar et al., 2022; Díaz-Puertas et al., 2023). Many alkaloids function as efflux-pump inhibitors (EPIs), targeting transport systems within the major facilitator superfamily and resistance-nodulation-division families.

Reserpine reverses Bmr-mediated multidrug resistance and inhibits drug transport in resistant strains (Gibbons and Udo, 2000; Wink et al., 2012; Díaz-Puertas et al., 2023). Berberine suppresses the MexXY-OprM efflux system in imipenem-resistant bacteria and demonstrates potent activity against MRSA, with reported MIC values of 32–128 mg/L and 90% growth inhibition at concentrations ≤64 mg/L (Yu et al., 2005; Laudadio et al., 2019; Díaz-Puertas et al., 2023). Structural diversity within alkaloid scaffolds has inspired the synthesis of derivatives incorporating indole, isoindole, imidazole, and oxazolidinone motifs, many of which exhibit broad-spectrum antibacterial activity (Kittakoop et al., 2014; Kumar et al., 2022; Díaz-Puertas et al., 2023).

Canthin-6-one isolated from *Allium neapolitanum* inhibits MRSA and tetracycline-resistant *S. aureus* (TRSA) with MIC values of 8 mg/L and 32 mg/L, respectively (O’Donnell and Gibbons, 2007; Kumar et al., 2022; Díaz-Puertas et al., 2023). Piperine, derived from Piper nigrum, exhibits anti-MRSA activity at 100 mg/L and enhances gentamicin efficacy through efflux-pump interference (Mohtar et al., 2009; Khameneh et al., 2015; Díaz-Puertas et al., 2023). Harmaline isolated from *Peganum harmala* reduces MRSA MIC values by four-to eight-fold, with effective concentrations ranging from 1.95 to 62.5 mg/L (Mohtar et al., 2009; Kumar et al., 2022; Díaz-Puertas et al., 2023). Evocarpine from *Tetradium ruticarpum* enhances oxacillin efficacy by sixteen-fold and demonstrates an MIC of 8 mg/L (Pan et al., 2014; Kumar et al., 2022; Díaz-Puertas et al., 2023).

Indirubin isolated from *Isatis tinctoria* exhibits activity against ciprofloxacin-resistant *S. aureus* with a reported MIC of 12.5 mg/L (Ponnusamy et al., 2010; Kumar et al., 2022; Díaz-Puertas et al., 2023). Additional alkaloids with significant activity against MRSA and TRSA include sanguinarine, tetrandrine, tryptanthrin, and galegine (Williams et al., 2010; Coqueiro et al., 2014; Costa et al., 2017). Indole-3-carbinol derived from cruciferous vegetables also demonstrates antibacterial activity against MDR-SA and enhances the efficacy of other antimicrobial agents (Monte et al., 2014; Kumar et al., 2022; Díaz-Puertas et al., 2023).

**FIGURE 1:**
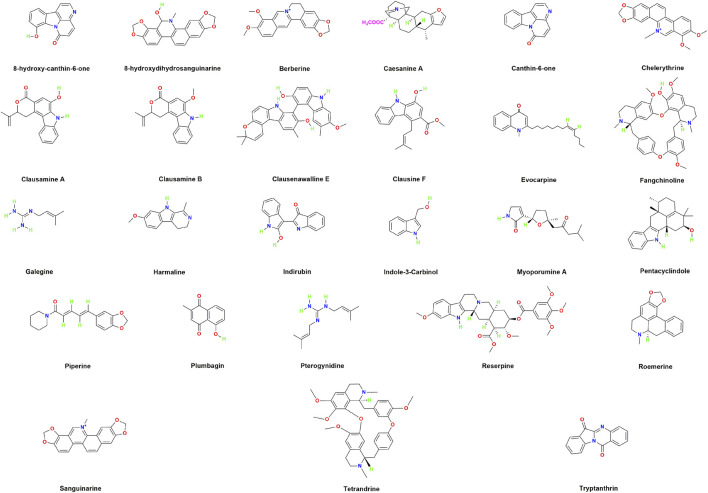
Chemical structures of potential alkaloid compounds with reported antimicrobial activity against MDR-SA.

The structural diversity of alkaloid scaffolds underpins their broad antibacterial activity against MDR-SA. Variations in heterocyclic ring systems, nitrogen positioning, and functional-group composition influence membrane interaction, efflux-pump inhibition, and bacterial target binding. Representative alkaloid structures with reported anti-MRSA activity are illustrated to highlight chemical features associated with antibacterial efficacy (Kittakoop et al., 2014; Díaz-Puertas et al., 2023). Figure 1 outlines representative alkaloid scaffolds with reported anti-MRSA activity, illustrating structural features associated with antibacterial efficacy.

### 2.2 Phenolic compounds and polyphenols

Phenolic compounds are characterized by hydroxyl groups attached to aromatic rings and represent a structurally diverse class of plant-derived metabolites with significant pharmacological activity against multidrug-resistant microorganisms. This group includes flavonoids, non-flavonoids, phenolic acids, and tannins, each contributing distinct chemical scaffolds and antibacterial mechanisms relevant to MDR-SA (Souza et al., 2019). Antibacterial activity arises from multiple interactions with bacterial systems, including disruption of membrane integrity, inhibition of virulence-associated enzymes and toxins, and suppression of biofilm formation, thereby positioning phenolics as promising natural antimicrobial agents (Miklasińska-Majdanik et al., 2018; Makarewicz et al., 2021).

Aspidinol isolated from *Dryopteris fragrans* demonstrates potent anti-MRSA activity, with MIC values ranging from 0.5 to 2 mg/L, and significantly improves survival outcomes in MRSA-infected murine models, achieving an 80% survival rate at a dose of 25 mg/kg (Hua et al., 2018). Galangin derived from *Alpinia officinarum* exhibits antibacterial activity against penicillin-resistant *S. aureus* (PRSA) with reported MIC values of 100–300 mg/L (Eumkeb et al., 2010). Hyperforin demonstrates strong activity against both MRSA and EMRSA at concentrations in the range of 0.5–2 mg/L (Schiavone et al., 2013). α-Mangostin from *Garcinia mangostana* also exhibits antibacterial efficacy, with MIC values between 1.57 and 12.5 mg/L against MRSA strains (Iinuma et al., 1996).

Isovalerylshikonin isolated from *Arnebia euchroma* suppresses antimicrobial resistance by targeting the MsrA efflux pump in MRSA, thereby enhancing intracellular drug retention (He et al., 2019). Rutin, when combined with carbon dots, exhibits antibacterial activity with an MIC of 32 mg/L against MRSA, highlighting the potential of phenolic compounds within hybrid nanoscale systems (Lang et al., 2024).

**FIGURE 2:**
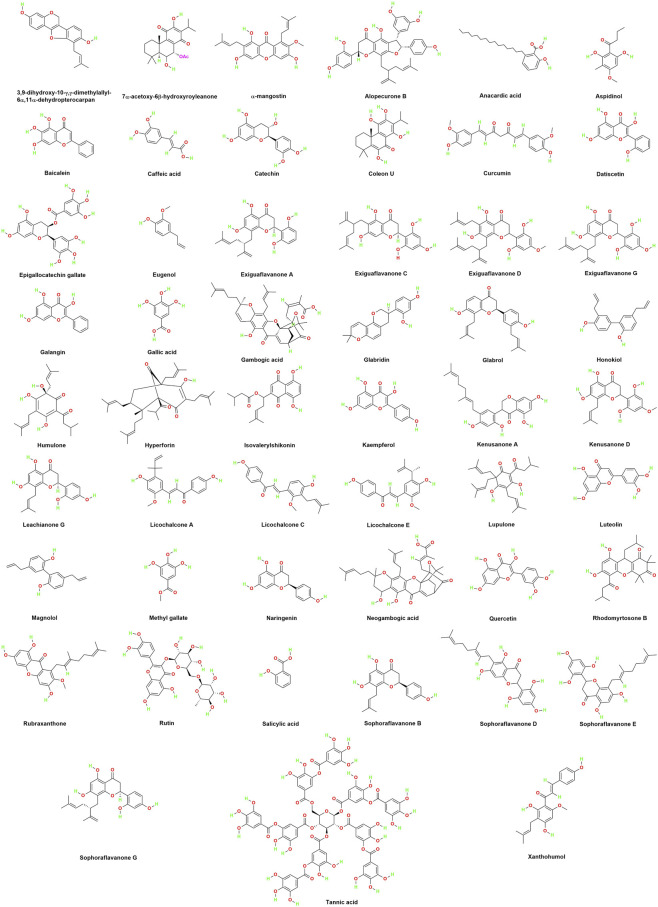
Chemical structures of potential phenolic compounds with reported antimicrobial activity against MDR-SA.

Additional phenolic compounds demonstrate broad antibacterial activity against MRSA, including gallic acid, catechin, quercetin, caffeic acid, eugenol, kaempferol, and epigallocatechin gallate (Xu and Lee, 2001; Luís et al., 2014; Yadav et al., 2015; Randhawa et al., 2016; Sinsinwar and Vadivel, 2020; Knidel et al., 2021). Baicalein exhibits activity against ciprofloxacin-resistant *S. aureus*, with MIC values ranging from 64 to 256 mg/L (Chan et al., 2011). Curcumin, glabridin, humulone, salicylic acid, and tannic acid demonstrate antibacterial effects across multiple MDR-SA strains, acting through combined mechanisms that include membrane disruption, metabolic interference, efflux modulation, and virulence suppression (Tsuchiya et al., 1996; Monte et al., 2014; Bogdanova et al., 2018; Kırmusaoğlu, 2019; Pinheiro et al., 2022). Figure 2 displays representative phenolic and polyphenolic structures with documented anti-MRSA activity, illustrating chemical motifs linked to antibacterial potency.

The structural diversity and multitarget mechanisms of phenolic compounds highlight their potential as effective antibacterial agents against MDR-SA. Their ability to disrupt membranes, inhibit virulence pathways, suppress efflux activity, and interfere with biofilm formation provides a strong mechanistic foundation for phenolic-based therapeutic strategies. The chemical diversity of phenolic and polyphenolic scaffolds contributes directly to variation in antibacterial potency and mechanism of action. Differences in hydroxylation patterns, ring conjugation, and molecular size influence membrane affinity, redox activity, and virulence modulation. Representative phenolic compounds with reported anti-MRSA activity are illustrated to highlight structural features associated with antibacterial efficacy (Makarewicz et al., 2021; Díaz-Puertas et al., 2023).

### 2.3 Terpenoids

Terpenoids constitute a major class of bioactive natural products derived from isoprene units and encompass monoterpenes, sesquiterpenes, diterpenes, and related derivatives. Their extensive structural diversity and frequent incorporation of oxygenated functional groups contribute to broad antibacterial activity against multidrug-resistant pathogens. The lipophilic nature of terpenoids facilitates interaction with microbial cell envelopes, resulting in membrane destabilization, increased permeability, and loss of cytoplasmic integrity (Trindade et al., 2015; Sil et al., 2020; Sharma et al., 2020; Musayeib et al., 2022). Antibacterial mechanisms include membrane disruption, inhibition of essential enzymes, interference with protein synthesis, induction of reactive oxygen species, and modulation of quorum-sensing and adhesion pathways (Mahizan et al., 2019; Adoui and Lahouel, 2019).

Guaianolide, a sesquiterpene lactone isolated from *Artemisia gilvescens*, demonstrates potent anti-MRSA activity with a reported MIC of 1.95 mg/L (Elmaidomy et al., 2022). Horminone, a diterpene quinone derived from *Salvia deserta*, exhibits antibacterial activity against MRSA with MIC values ranging from 7.81 to 15.63 mg/L (Gaspar-Marques et al., 2006). Mansonone F, a sesquiterpene isolated from *Ulmus davidiana* var. *japonica*, exhibits antibacterial activity against nineteen MRSA strains, with a reported MIC of 2 mg/mL (Park et al., 2018). In comparison, totarol from *Podocarpus nagi* demonstrates strong antibacterial activity against MRSA strains, with MIC values ranging from 1.56 to 3.12 mg/L (Yu et al., 2015).

Additional terpenoid-derived compounds with activity against MRSA include 18β-glycyrrhetinic acid, 6-*O*-isobutyroylplenolin, betulin, betulinaldehyde, betulinic acid, dehydroepingaione, dehydroleucodine, dehydromyoporone, lupeol, xanthatin, zerumbol, and α-amyrin (Sato et al., 1997; Taylor and Towers, 1998; Ordóñez et al., 2011; Chung et al., 2014; Wang et al., 2015; Wang et al., 2016; Long et al., 2013; Dong et al., 2018; Siddique et al., 2019). Compounds such as dehydroabietic acid, isopimaric acid, and sabinene also demonstrate antibacterial activity against MDR-SA strains (Smith et al., 2005; Leandro et al., 2014; Matias et al., 2016).

**FIGURE 3:**
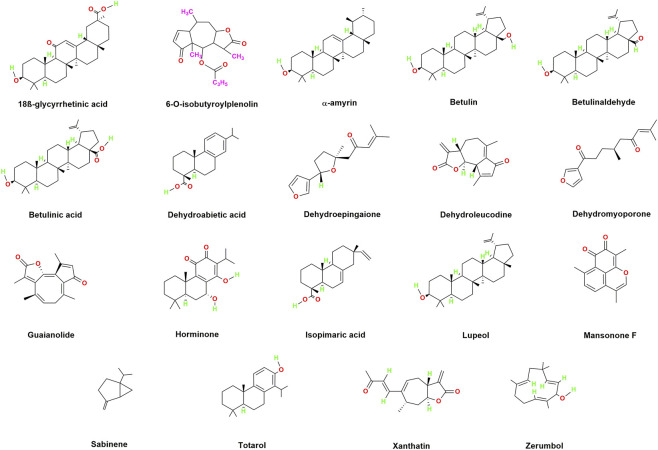
Chemical structures of potential terpenoids with reported antimicrobial activity against MDR-SA.

The structural diversity and multitarget mechanisms of terpenoids highlight their potential as broad-spectrum antibacterial agents against resistant *S. aureus*. Their demonstrated efficacy across multiple MRSA lineages supports continued investigation into terpenoid-based therapeutic applications. Structural variation across terpenoid scaffolds contributes directly to differences in antibacterial potency and mechanism of action. Modulation of ring systems, degree of oxidation, and substituent positioning influences membrane affinity, intracellular target engagement, and oxidative-stress induction. Representative terpenoid structures with reported anti-MRSA activity are illustrated to highlight chemical features associated with antibacterial efficacy (Mahizan et al., 2019; Musayeib et al., 2022). Figure 3 presents representative terpenoid scaffolds with reported activity against MDR-SA, illustrating structural elements associated with antibacterial mechanisms.

### 2.4 Coumarins

Coumarins form a prominent class of heterocyclic benzopyrone derivatives widely distributed in plant species and recognized for diverse pharmacological activities, including antibacterial, antiviral, antioxidant, anti-inflammatory, and antitumor effects. Their antibacterial activity is primarily attributed to inhibition of bacterial DNA gyrase, a critical enzyme involved in DNA supercoiling, replication, and cell division (Garg et al., 2020). This mechanism confers broad activity against MDR-SA strains.

A highly active coumarin, 5,7-dihydroxy-8-(2-methylbutanoyl)-6-(3-methylbut-2-enyl)-4-phenyl-2H-chromen-2-one, isolated from *Mesua ferrea*, demonstrates potent antibacterial activity against methicillin-resistant *S. aureus* (MRSA) and fluoroquinolone-resistant *S. aureus* (FRSA), with reported MIC values of 6.25 mg/L and 12.5 mg/L, respectively (Roy et al., 2013). Additional coumarins, including 5-geranyloxy-7-methoxycoumarin, artanin, isopimpinellin, and phellopterin, exhibit notable antibacterial activity against MRSA strains such as MRSA098, with MIC values ranging from 8 to 64 mg/L (Zuo et al., 2016). These compounds exert antibacterial effects through combined mechanisms that include DNA gyrase inhibition, membrane destabilization, and interference with resistance-associated pathways.

**FIGURE 4:**
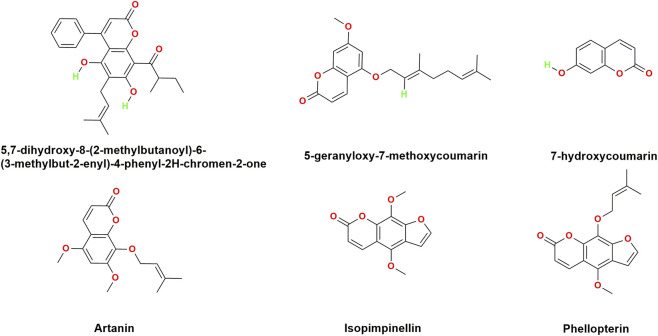
Chemical structures of potential coumarins with reported antimicrobial activity against MDR-SA.

The structural diversity and mechanistic breadth of coumarins highlight their potential as natural antibacterial agents against MDR-SA. Their demonstrated efficacy against resistant *S. aureus* lineages supports continued investigation into coumarin-based therapeutic strategies. Variation within coumarin scaffolds influences antibacterial potency and spectrum of activity. Substituent position, degree of prenylation, and ring functionalization modulate target interaction, membrane affinity, and enzyme inhibition. Representative coumarin structures with reported antibacterial activity against MDR-SA are presented to illustrate structure-activity relationships relevant to antimicrobial efficacy (Zuo et al., 2016; Garg et al., 2020). Figure 4 outlines representative coumarin structures with anti-MRSA activity, highlighting substituent patterns relevant to antibacterial performance.

### 2.5 Cannabinoids

Cannabinoids comprise a structurally diverse group of bioactive compounds that include phytocannabinoids, endocannabinoids, and synthetic analogues. These molecules are well recognized for psychoactive, anticonvulsive, analgesic, and neuroprotective properties, mediated through cannabinoid receptor-dependent pathways involving CB1 and CB2, as well as receptor-independent mechanisms. Beyond neuromodulatory effects, several cannabinoids exhibit antibacterial activity against MDR-SA, expanding their therapeutic relevance beyond neurological applications (Appendino et al., 2008).

Cannabichromene, cannabidiol, cannabigerol, and cannabinol isolated from *Cannabis sativa* L. demonstrate strong antibacterial activity against multiple MRSA strains. Reported minimum inhibitory concentration values range from 1 to 2 mg/L for cannabichromene, 0.5–1 mg/L for cannabidiol, 1–2 mg/L for cannabigerol, and approximately 1 mg/L for cannabinol (Appendino et al., 2008). These findings indicate substantial inhibitory capacity across structurally distinct phytocannabinoids and highlight their potential as candidates for the development of alternative therapeutic strategies targeting resistant *S. aureus* infections.

**FIGURE 5:**
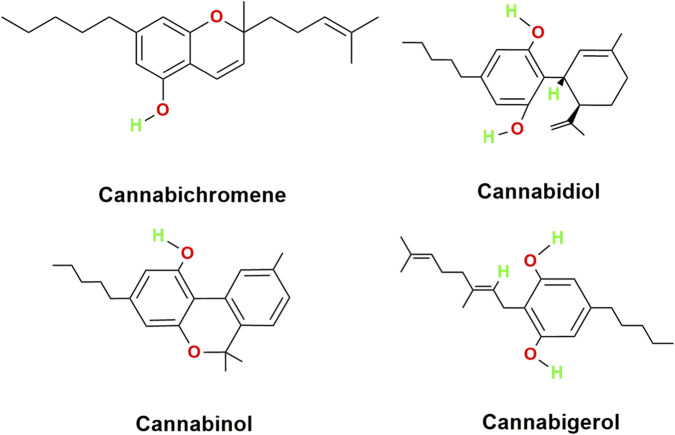
Chemical structures of potential cannabinoid compounds with reported antimicrobial activity against MDR-SA.

The demonstrated antibacterial activity of cannabinoids reinforces their relevance as natural antimicrobial agents against MDR-SA. Their efficacy across multiple resistant strains supports continued investigation into cannabinoid-bacteria interactions, structure-activity relationships, and potential integration into novel antimicrobial platforms (Appendino et al., 2008). Structural variation among cannabinoid scaffolds contributes to differences in antibacterial potency and spectrum of activity. Modulation of aromatic substitution patterns, alkyl side-chain length, and hydroxyl-group distribution influences lipophilicity, membrane interaction, and bacterial target engagement. Representative cannabinoid structures with reported anti-MRSA activity are illustrated to highlight chemical features associated with antibacterial efficacy (Appendino et al., 2008). Figure 5 showcases representative cannabinoid scaffolds with reported anti-MRSA activity, emphasizing structural determinants of antibacterial efficacy. Table 1 summarises phytochemicals with reported antibacterial activity against MDR-SA, grouped by chemical class and supported by MIC values.

**TABLE 1:** Phytochemicals showing potential antibacterial efficacy against MDR-SA.

Chemical class	Phytochemical	Bacterial species	MIC value	References
Alkaloids	8-Hydroxy-canthin-6-one	TetRSA and MRSA	8 and 64 mg/L, respectively	O’Donnell and Gibbons (2007)
	8-Hydroxydihydrosanguinarine	MRSA	1.95mg/L	Zuo et al. (2008)
	Berberine	MRSA	32–128mg/L	Yu et al. (2005)
	Caesanine A	MRSA	12.5mg/L	Zhang et al. (2013)
	Canthin-6-one	TetRSA and MRSA	8 and 32mg/L, respectively	O’Donnell and Gibbons (2007)
	Chelerythrine	MRSA	2–4mg/L	Wang et al. (2021)
	Clausamine A	MRSA	8mg/L	Maneerat et al. (2012)
	Clausamine B	MRSA	0.25mg/L	Maneerat et al. (2012)
	Clausenawalline E	MRSA	8mg/L	Maneerat et al. (2012)
	Clausine F	MRSA	4mg/L	Maneerat et al. (2012)
	Evocarpine	MRSA strain 33591	8mg/L	Ponnusamy et al. (2010)
	Fangchinoline	MRSA strain 13366	160mg/L	Fu et al. (2017)
	Galegine	Epidemic-MRSA-15 strain	4 mg/L	Coqueiro et al. (2014)
	Harmaline	Two clinical strains of MRSA	MIC at 1.95 and 62.5mg/L, respectively	Mohtar et al. (2009)
	Indirubin	Ciprofloxacin-resistant *S. aureus*	12.5mg/L	Ponnusamy et al. (2010)
	Indole-3-carbinol	MDR strain of *S. aureus*	400mg/L	Monte et al. (2014)
	Myoporumine A	MRSA	6.25mg/L	Dong et al. (2018)
	Pentacyclindole	MRSA-105 strain	8mg/L	Lopez and Osada (2020)
	Piperine	MRSA	100mg/L	Khameneh et al. (2015)
	Plumbagin	MRSA	4–8mg/L	Periasamy et al. (2019)
	Pterogynidine	Epidemic-MRSA-15 strain	4mg/L	Coqueiro et al. (2014)
	Reserpine	MRSA	100mg/L	Randhawa et al. (2016)
	Roemerine	MRSA	32–64mg/L	Yin et al. (2015)
	Sanguinarine	MRSA	3.12–6.25mg/L	Obiang-Obounou et al. (2011)
	Tetrandrine	MRSA strain 13366	80mg/L	Fu et al. (2017)
	Tryptanthrin	MRSA strain BMB9393	62.5mg/L	Costa et al. (2017)
Cannabinoids	Cannabichromene	MRSA XU212 strain	1–2mg/L	Appendino et al. (2008)
	Cannabidiol	MRSA XU212 strain	0.5–1mg/L	Appendino et al. (2008)
	Cannabigerol	MRSA XU212 strain	1–2mg/L	Appendino et al. (2008)
	Cannabinol	MRSA XU212 strain	1mg/L	Appendino et al. (2008)
Coumarin	5,7-dihydroxy-8-(2-methylbutanoyl)-6-(3-methylbut-2-enyl)-4-phenyl-2*H*-chromen-2-one	Fluoroquinolone-resistant mutant of SA-1199 strain and MRSA strain 831	12.5 and 6.25mg/L, respectively	Roy et al. (2013)
	5-geranyloxy-7-methoxycoumarin	MRSA	8–64mg/L	Zuo et al. (2016)
​	7-hydroxycoumarin	MDR strain of *S. aureus*	200mg/L	Monte et al. (2014)
	Artanin	MRSA098	8–64mg/L	Zuo et al. (2016)
	Isopimpinellin	MRSA	32–64mg/L	Zuo et al. (2016)
	Phellopterin	MRSA	8–32mg/L	Zuo et al. (2016)
Phenols	3,9-Dihyroxy- 10-γ, γ-dimethylallyl- 6α,11α- dehydropterocarpan	MRSA G47 strain	12.5mg/L	Tanaka et al. (2004)
	7*α*-acetoxy-6*β*-hydroxyroyleanone	MRSA	3.91–15.63mg/mL	Gaspar-Marques et al. (2006)
	Alopecurone B	MRSA	3.13–6.25mg/L	Sato et al. (1995)
	Anacardic acid	MRSA	>100μmol/L	Saedtler et al. (2020)
	Aspidinol	Clinical isolate of MRSA strain and MRSA (*in vivo*)	0.5–2mg/L. An *in vivo* study of aspidinol against an MRSA strain in mice found that 80% of mice survived for 5 days at a dose of 25 mg/kg	Hua et al. (2018)
	Baicalein	Ciprofloxacin-resistant *S. aureus*	64–256mg/L	Chan et al. (2011)
	Caffeic acid	MRSA	62.5–250mg/L	Luís et al. (2014)
	Catechin	MRSA	78.1mg/L	Sinsinwar and Vadivel (2020)
	Coleon U	MRSA	0.98–31.25mg/mL	Gaspar-Marques et al. (2006)
	Curcumin	MDR strains of *S. aureus*	125–250mg/L	Mun et al. (2013)
	Datiscetin	MRSA strain 9247922	512mg/L	Xu and Lee (2001)
	Epigallocatechin gallate	MRSA	7.81–62.5mg/L	Knidel et al. (2021)
	Eugenol	MRSA (*in vitro* and *in vivo*)	0.1%–0.4%. *In vivo* analysis showed that sub-MIC eugenol inhibited growth by 88% in the rat middle ear	Yadav et al. (2015)
	Exiguaflavanone A	MRSA	6.25mg/L	Tsuchiya et al. (1996)
	Exiguaflavanone C	MRSA	12.5mg/L	Tsuchiya et al. (1996)
	Exiguaflavanone D	MRSA	3.13–6.25mg/L	Tsuchiya et al. (1996)
	Exiguaflavanone G	MRSA	12.5mg/L	Tsuchiya et al. (1996)
	Galangin	Penicillin-resistant *S. aureus*	100–300mg/L	Eumkeb et al. (2010)
	Gallic acid	MRSA	4mg/L	Luís et al. (2014)
	Gambogic acid	MRSA strains	0.5–4mg/L	Hua et al. (2019)
	Glabridin	MDR strain of *S. aureus*	3.12–25mg/L	Singh et al. (2015)
	Glabrol	MRSA strain T144	2mg/L	Wu et al. (2019)
	Honokiol	MRSA	16–32mg/L	Zuo et al. (2015)
	Humulone	MDR strain of *S. aureus*	15mg/L	Bogdanova et al. (2018)
	Hyperforin	Clinical MRSA strains XU212 and Ig5 and EMRSA-15 strain	0.5–2mg/L	Schiavone et al. (2013)
	Isovalerylshikonin	MRSA strain RN4220	16mg/L	He et al. (2019)
	Kaempferol	Fluoroquinolone-resistant *S. aureus* and MRSA	250mg/L and 125mg/L, respectively	Randhawa et al. (2016)
	Kenusanone A	MRSA	6.25–12.5mg/L	Tsuchiya et al. (1996)
​	Kenusanone D	MRSA	3.13–12.5mg/L	Tsuchiya et al. (1996)
	Leachianone G	MRSA	12.5mg/L	Tsuchiya et al. (1996)
	Licochalcone A	MRSA strain T144	4mg/L	Wu et al. (2019)
	Licochalcone C	MRSA strain T144	4mg/L	Wu et al. (2019)
	Licochalcone E	MRSA strain T144	T4mg/L	Wu et al. (2019)
	Lupulone	MRSA	0.5mg/L	Bogdanova et al. (2018)
	Luteolin	MRSA strain 9247922	512mg/L	Xu and Lee (2001)
	Magnolol	MRSA	8–64mg/L	Zuo et al. (2015)
	Methyl gallate	MRSA strain ATCC33591	250mg/L	Chew et al. (2018)
	Naringenin	MRSA	200–400mg/L	Tsuchiya et al. (1996)
	Neogambogic acid	MRSA ATCC33591 strain	0.5–4mg/L	Hua et al. (2019)
	Quercetin	MRSA strain 9247922	256mg/L	Xu and Lee (2001)
	Rhodomyrtosone B	MRSA (*In vitro* and *In vivo*)	0.62–1.25mg/L. An *in vivo* study in mice showed that it attenuated skin ulceration in a murine model of MRSA infection at a single dose of 40µg per mouse	Zhao et al. (2019)
	Rubraxanthone	MRSA	0.31–1.25mg/L	Iinuma et al. (1996)
	Rutin	MRSA	32mg/L	Lang et al. (2024)
	Salicylic acid	MDR strain of *S. aureus*	1600mg/L	Monte et al. (2014)
	Sophoraflavanone B	MRSA	15.60–31.25mg/L	Mun et al. (2014)
	Sophoraflavanone D	MRSA	3.13–12.5mg/L	Tsuchiya et al. (1996)
	Sophoraflavanone E	MRSA	6.25–12.5mg/L	Tsuchiya et al. (1996)
	Sophoraflavanone G	MRSA	3.13–6.25mg/L	Tsuchiya et al. (1996)
	Tannic acid	Five MDR strains of *S. aureus*	4–256mg/L by the authors	Kırmusaoğlu (2019)
	Xanthohumol	MRSA	4mg/L	Bogdanova et al. (2018)
	α-mangostin	MRSA	1.57–12.5mg/L	Iinuma et al. (1996)
Terpenoids	18β-glycyrrhetinic acid	MRSA USA400 strain	60mg/L	Long et al. (2013)
	6-*O*-isobutyroylplenolin	MRSA	300mg/L	Taylor and Towers (1998)
	Betulin	MRSA ATCC43300 strain	128mg/L	Wang et al. (2016)
	Betulinaldehyde	MRSA ATCC43300 strain	8–512mg/L	Chung et al. (2014)
	Betulinic acid	MRSA	4–64mg/L	Chung et al. (2014)
	Dehydroabietic acid	MDR strains of *S. aureus*	6.25 and 50mg/L	Leandro et al. (2014)
	Dehydroepingaione	MRSA	25mg/L	Costa et al. (2017)
	Dehydroleucodine	MRSA	49–147mg/L	Ordóñez et al. (2011)
	Dehydromyoporone	MRSA	50mg/L	Dong et al. (2018)
	Guaianolide	MRSA	1.95mg/L	Elmaidomy et al. (2022)
	Horminone	MRSA	7.81–15.63mg/L	Gaspar-Marques et al. (2006)
​	Isopimaric acid	MDR strains of *S. aureus*	32–64mg/L	Smith et al. (2005)
	Lupeol	MRSA	4mg/L	Wang et al. (2016)
	Mansonone F	MRSA strain	2mg/L	Park et al. (2018)
	Sabinene	MDR strains of *S. aureus*	1024mg/L	Matias et al. (2016)
	Totarol	MRSA strains NRS-1, NRS-70, NRS-100, NRS-108 and NRS-271	1.56–3.12mg/L	Yu et al. (2015)
	Xanthatin	MRSA	7.8–15.6mg/L	Sato et al. (1997)
	Zerumbol	MRSA	32–128mg/L	Siddique et al. (2019)
	α-Amyrin	MRSA	2–64mg/L	Chung et al. (2014)

## 3 Advancement of nanotechnology platforms to counter MDR *S. aureus* infections

Nanotechnology provides a strategic framework for overcoming key limitations of conventional antibiotics, particularly poor cellular uptake, restricted penetration across bacterial membranes, and limited intracellular bioavailability (Mba and Nweze, 2021). Nanoparticles (NPs) enhance intracellular delivery through endocytosis-mediated uptake and direct interactions with membrane lipids, thereby improving internalization and therapeutic exposure. Encapsulation within nanosystems offers physical protection to antimicrobial agents, reducing susceptibility to enzymatic degradation and mitigating efflux pump-mediated resistance (Huh and Kwon, 2011; Wang et al., 2017). In addition to delivery enhancement, many nanosystems exhibit intrinsic antibacterial activity through combined mechanisms that include disruption of bacterial cell walls, inhibition of biofilm formation, induction of reactive oxygen species (ROS), and intracellular DNA and protein damage (Wang et al., 2017; Baptista et al., 2018). These properties establish nanotechnology as a transformative platform for managing persistent MDR-SA infections.

**FIGURE 6:**
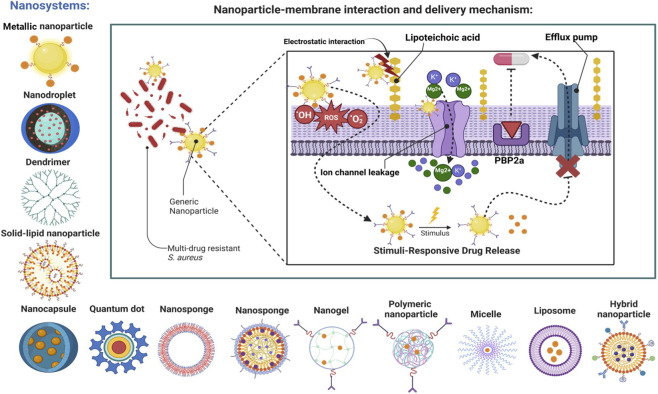
Recent nanosystems designed to combat MDR-SA, highlighting nanoparticle-membrane interactions and drug-delivery mechanisms.

Recent advances in nanosystem engineering have led to platforms that interact directly with bacterial surfaces while simultaneously serving as drug-delivery vehicles. Such dual functionality enables both physical destabilization of bacterial membranes and improved accumulation of antimicrobial agents at infection sites. Figure 6 summarises representative nanosystems developed for combating MDR-SA, highlighting nanoparticle-membrane interactions and key drug-delivery mechanisms relevant to antimicrobial efficacy (Wang et al., 2017; Mba and Nweze, 2021).

Nanoparticles exert broad-spectrum antibacterial activity against MDR-SA through synergistic physicochemical mechanisms that circumvent canonical resistance pathways. These mechanisms include ROS-mediated oxidative stress, disruption of membrane integrity and fluidity, pore formation, and electrostatic destabilization through interactions with negatively charged cell wall components such as lipoteichoic acid, ultimately leading to cytoplasmic leakage and cell lysis (Wang et al., 2017; Baptista et al., 2018). Concurrently, NPs perturb ionic homeostasis by inducing potassium and magnesium efflux, impairing protein function, and increasing membrane permeability. Nanosystems further bypass efflux pump-mediated resistance by facilitating intracellular delivery of antimicrobial cargo or through direct interference with efflux activity (Huh and Kwon, 2011). These coordinated mechanisms underpin the development of diverse nanoplatforms engineered to enhance antibacterial efficacy, biofilm penetration, and therapeutic durability against MDR-SA infections.

### 3.1 Liposomes

Liposomes, owing to their biomimetic phospholipid bilayer structure, enable dual encapsulation of hydrophilic and hydrophobic therapeutics and efficient intracellular delivery (Akbarzadeh et al., 2013; Bozzuto and Molinari, 2015). Advanced systems such as TLR4-targeted, hyaluronic-acid-modified vancomycin-loaded liposomes (∼121 nm; 49.97% encapsulation efficiency) exhibited a fivefold increase in antibacterial and antibiofilm activity compared with free drug, attributed to enhanced stability and targeted delivery (Ismail et al., 2024). Integration of vancomycin-loaded liposomes within PLGA-PEG-PLGA hydrogels co-delivering DNase I enabled sequential release (77.2% DNase I in 72 h; 82.6% vancomycin over 14 days), effectively disrupting MRSA biofilms and promoting bone regeneration (Li et al., 2023). Oral tetraether-lipid-stabilized liposomes functionalized with cell-penetrating peptides for delivery of a vancomycin derivative (FU002) further demonstrated significant reduction in hepatic MRSA burden at an MIC of 1 mg/L, underscoring translational potential (Werner et al., 2024).

### 3.2 Solid lipid nanoparticles (SLNPs)

Solid lipid nanoparticles (SLNPs) provide enhanced physicochemical stability, controlled release, and responsiveness to infection-specific microenvironments (Naseri et al., 2015; Scioli Montoto et al., 2020). Enzyme-responsive vancomycin-loaded SLNPs (VCM-AS-SLNs; ∼102.2 nm) achieved superior antibacterial activity (MIC 1.56 mg/L vs. 3.125 mg/L for free drug), complete bacterial eradication within 12 h at elevated concentrations, and fivefold higher biofilm removal *via* lipase-triggered release (∼80%) (Mohammed et al., 2023). DNase I- or Tween-80-modified gentamicin-loaded SLNPs (∼293 nm; 16.85% encapsulation efficiency) demonstrated enhanced antibiofilm efficacy (86.28% vs. 65.34% for free drug) with MIC/MBC values of 3.14/6.28 mg/L (Maurya et al., 2024).

### 3.3 Micelles

Micellar systems, formed through amphiphilic self-assembly, enable targeted delivery and biofilm disruption by modulating bacterial surface hydrophobicity and facilitating penetration (Torchilin, 2007; Perumal et al., 2022). Stimuli-responsive micelles, including fluorinated and light-activated systems, enhance ROS-mediated killing and biofilm eradication (Wan et al., 2023; Lu et al., 2025; Wang et al., 2025). Mercaptosuccinic-acid-decorated micelles (DCSMs) reduced ciprofloxacin MIC fourfold (6.25 mg/L at pH 5.5 vs. 25 mg/L) and achieved ∼85% biofilm inhibition, whereas Magainin II-modified pH-sensitive micelles co-loaded with azithromycin and luteolin (∼40.17 nm; encapsulation efficiencies 93%–94%) demonstrated potent antibacterial activity (MIC 0.625 mg/L) and 86.5% biofilm inhibition (Yang L. et al., 2024; Wang et al., 2025).

### 3.4 Nanodroplets

Nanodroplet-based systems introduce a complementary physical modality *via* ultrasound-triggered cavitation, enhancing antibiotic penetration and mechanically disrupting biofilms (Argenziano et al., 2017; Banche et al., 2022). High-pressure nanodroplets (∼100 nm) induced peptidoglycan leakage even at 5 cm under 75 MPa, while phase-shift acoustic nanodroplets combined with vancomycin reduced residual biofilm to 7.43% following ultrasound activation, confirming synergistic efficacy (Guo et al., 2017; Tamura et al., 2024).

### 3.5 Dendrimers

Dendrimers, defined by their highly branched architecture and tunable surface functionality, facilitate efficient drug encapsulation and strong electrostatic interactions with bacterial membranes (Tülü and Erturk, 2012). Cationic V-3-mPEA dendrimers (∼52.48 nm) achieved a 16-fold reduction in MIC (0.98 mg/L vs. 15.65 mg/L for vancomycin) and ∼90% reduction in biofilm biomass through membrane disruption and intracellular leakage (Omolo et al., 2018). Nitric-oxide-releasing *N*-diazeniumdiolate-functionalized PPI dendrimers further demonstrated concentration-dependent antibacterial activity, with G5 variants achieving 3-log MRSA reduction at 2.5–5 µM (Sun et al., 2012).

### 3.6 Polymeric nanoparticles (PNPs)

Polymeric nanoparticles (PNPs), particularly those based on PLGA and PLA, offer high structural integrity, biodegradability, and scalable drug delivery (Cano et al., 2020; Spirescu et al., 2021; Yu et al., 2023; Ali Khan et al., 2024; Naik et al., 2024). Biguanide-derived FTP-NPs produced *via* polyphenol-assisted aggregation exhibited markedly enhanced antibiofilm activity (up to 5.2 log_10_ reduction) and reduced biofilm thickness (3.6 µm vs. 12.8 µm for free polymer), alongside superior *in vivo* efficacy compared with vancomycin (Li et al., 2020). Neutrophil-targeted combinatorial nanosystems (NTCNs) further integrated immunomodulation, reducing TNF-α and IL-6 levels by ∼60% and bacterial load by 85%, outperforming conventional therapy (Chang et al., 2024).

### 3.7 Polymersomes

Polymersomes extend these capabilities through stimuli-responsive vesicular systems, enabling sustained release and multimodal therapy (Discher and Ahmed, 2006; Lee and Feijen, 2012; Zhang and Zhang, 2017). A copper-infused zinc-porphyrin polymersome (CU-PPS) induced localized heating (50.2 °C) and hydroxyl-radical generation, achieving 93% biofilm reduction and ∼80% bacterial killing under near-infrared irradiation, with complete wound closure *in vivo* within 14 days (Yang N. et al., 2024). Hyaluronic-acid-grafted oleylamine polymersomes maintained prolonged antibacterial activity, with consistent MIC values (1.95 mg/L) over 72 h, compared with rapid loss of efficacy for free vancomycin (Walvekar et al., 2019).

### 3.8 Nanogels

Nanogels, composed of crosslinked hydrophilic polymer networks, enable high drug-loading capacity and stimuli-responsive release (Coll Ferrer et al., 2014; Anooj et al., 2021; Keskin et al., 2021). Polypeptide nanogels synthesized *via* N-carboxyanhydride polymerization exhibited strong electrostatic binding (6.02 x 10^5^ M^-1^) and effective MRSA inhibition (MIC 4–16 mg/L), while multifunctional ROS-scavenging nanogels incorporating superoxide dismutase, catalase, and vancomycin achieved 55.1% bacterial inhibition and 92.26% wound healing in hyperglycaemic models (Wang et al., 2025).

### 3.9 Nanosponges

Nanosponges, particularly red-blood-cell (RBC)-membrane-coated systems, provide toxin-neutralizing and biomimetic delivery capabilities (Sherje et al., 2017; Ma et al., 2022; Song et al., 2023). RBC-coated nanosponges (∼100 nm; −28.7 mV) effectively sequestered α-haemolysin and achieved 100% survival in toxin-challenged mice at 28 mg/kg, while redox-responsive nanosponge systems reduced intracellular MRSA burden to <2 CFU per 100 macrophages and exhibited an MIC of 2.5 mg/L (Zagami et al., 2023). Cyclodextrin-based nanosponges loaded with porphyrins further demonstrated photodynamic antibiofilm activity (Zagami et al., 2023).

### 3.10 Quantum dots (QDs)

Quantum dots (QDs), owing to their unique optical and redox properties, provide both diagnostic and therapeutic functionality. Nitrogen-doped QDs achieved >99% MRSA eradication, while graphene QD-Ag hybrids (MIC 11.4 mg/L) and MoS_2_ QDs (MIC 0.65 mg/L) exhibited enhanced antibacterial activity *via* ROS generation, glutathione depletion (77%), and membrane depolarisation, alongside low cytotoxicity and accelerated wound healing (Zhong et al., 2020; Ali and De, 2023).

### 3.11 Metallic nanoparticles and hybrid systems

Metallic nanosystems, encompassing metal, metal-oxide, and hybrid nanocomposites, exert antibacterial effects through ROS generation, membrane disruption, DNA damage, and metabolic inhibition (Jan et al., 2014; Perelshtein et al., 2015; Stanić and Tanasković, 2020; Shabatina et al., 2023; Sklodowski et al., 2023). Ag@ZnO-incorporated PVA/PVP hydrogels demonstrated strong antibacterial activity and wound healing, while Ag/CaO nanocomposites (MIC 25 mg/L; MBC 150 mg/L) achieved up to 85% biofilm reduction with reduced cytotoxicity (Khan et al., 2020; Khan et al., 2021). Hybrid GA@TiO_2_ nanoparticles embedded in GO/CMCh hydrogels further enhanced antibacterial efficacy (MIC 75 mg/L vs. 312.5 mg/L for TiO_2_ alone) (Salama et al., 2024). Additional metallic systems, including Au, Zn, Cu, and ZnO nanoparticles, reinforce broad-spectrum activity against MDR-SA (Modi et al., 2023).

### 3.12 Emerging nanocomposites and hybrid platforms

Emerging nanosystems such as ceramic nanocomposites (3C-SiC@g-C_3_N_4_; MIC 2 mg/mL), curcumin-loaded gold nanocapsules, essential-oil nanocapsules (MIC 31.25 mL/L), Ag-containing TiO_2_ nanocapsules with sustained release (>70 days), biogenic Ag-Au nanoparticles with quercetin (20 mg/L), and selenium-chitosan hybrids (MIC/MBC 3.9 mg/L) highlight the expanding diversity of nanotherapeutic strategies (Bhatia and Banerjee, 2020; Hérault et al., 2020; Baig et al., 2021; Thorat et al., 2021; Bouaouina et al., 2022; Gamal et al., 2024). Collectively, these interconnected nanosystems demonstrate a paradigm shift towards multifunctional, mechanism-driven platforms capable of overcoming resistance, disrupting biofilms, and enabling sustained, targeted therapy for MDR-SA infections.

## 4 Phytochemical-based nanoparticles: Advanced approaches against MDR *S*. *aureus*

### 4.1 Pharmacokinetics and formulation advantages

The rising incidence of antibiotic resistance necessitates the development of novel antimicrobial strategies that enhance therapeutic efficacy while minimizing adverse effects. Phytochemical-based nanoparticles (phyto-NPs), which integrate bioactive plant-derived compounds with nanoscale delivery systems, have emerged as a promising antibacterial platform. These systems leverage the inherent antimicrobial properties of phytochemicals while improving their solubility, stability, bioavailability, and intracellular accumulation. Beyond these formulation advantages, the therapeutic performance of phyto-NPs is closely linked to their modified pharmacokinetic profile. Free phytochemicals are often constrained by low aqueous solubility, limited permeability, and rapid presystemic metabolism, resulting in poor systemic exposure. Nanoencapsulation overcomes these barriers by enhancing apparent solubility and facilitating epithelial transport *via* endocytic pathways, thereby improving bioavailability. Following systemic absorption, nanoparticle size, surface charge, and interfacial properties govern protein corona formation, which in turn modulates circulation time, immune recognition, and biodistribution. Optimized phyto-NPs can reduce rapid clearance, lower required doses, prolong circulation, and enable preferential accumulation at infection sites through personalized delivery, particularly under inflammatory conditions characteristic of MDR-SA infections (Díaz-Puertas et al., 2023; Guedes et al., 2024).

Encapsulation improves phytochemical stability by protecting bioactive compounds from premature degradation, thereby prolonging circulation time and sustaining therapeutic concentrations (Devi et al., 2014). These effects are further reinforced by controlled release kinetics, which sustain drug levels within the therapeutic window while limiting systemic toxicity. Clearance pathways are dependent on carrier composition and size, with biodegradable systems supporting efficient elimination and reduced long-term accumulation. For example, apigenin-loaded liposomes enhanced membrane interaction and intracellular accumulation, reducing the MIC from 32 mg/L (free apigenin) to 8 mg/L (Banerjee et al., 2015). Essential-oil nanoformulations also demonstrate improved stability and permeability: stearic-acid solid-lipid nanoparticles loaded with *Eugenia caryophyllata* essential oil reduced the MIC from 0.5 mg/L to 0.25 mg/L (Fazly et al., 2018). The physicochemical properties of nanoparticles, particularly size and shape, critically influence antibacterial performance. Smaller nanoparticles exhibit a larger surface-area-to-volume ratio, enabling stronger bactericidal effects (Kvitek et al., 2008). Antibacterial activity declines with increasing particle size (Li K et al., 2013). Nanoparticles within the 5–15 nm range demonstrate enhanced antimicrobial efficacy due to improved membrane attachment, increased permeability, and accelerated cell death (Sondi and Salopek-Sondi, 2004). Shape also modulates antimicrobial potency, spherical silver nanoparticles derived from *Euphorbia hirta* L. exhibited potent anti-*S. aureus* activity with an MIC of 9 mg/L (Devi et al., 2014), while cubical silver nanoparticles outperformed spherical forms (Yousaf et al., 2020). Rod-shaped ZnO nanoparticles demonstrated superior antibacterial activity compared with plate-like structures (Ann et al., 2014; Jones et al., 2007). Collectively, these pharmacokinetic enhancements drive improved pharmacodynamic outcomes, notably increased intracellular delivery and enhanced penetration into bacterial biofilms, both of which are critical for overcoming resistance mechanisms in MDR-SA.

### 4.2 Mechanistic antibacterial activity of phyto-NPs

The multifaceted antibacterial mechanisms position phyto-NPs as robust candidates for combating MDR-SA. Phyto-NPs exert bactericidal activity through coordinated extracellular and intracellular mechanisms driven by the synergistic interplay between the nanocarrier and the bioactive phytochemical cargo. Extracellularly, nanoparticles adsorb onto the bacterial surface, where electrostatic interactions and surface reactivity disrupt membrane integrity and interfere with nutrient transport and signaling pathways. In Gram-positive bacteria such as *S. aureus*, the anionic peptidoglycan matrix promotes nanoparticle adhesion and facilitates localized accumulation, enhancing membrane destabilization (Sarwar et al., 2015). However, unlike conventional nanoparticles, phyto-NPs introduce an additional biochemical dimension. Surface-bound and released phytochemicals can directly interact with membrane lipids and proteins, altering fluidity, inducing lipid peroxidation, and inhibiting membrane-associated enzymes. Following membrane interaction, phyto-NPs can translocate into the cytoplasm *via* membrane perturbation or endocytic-like processes. Intracellularly, their activity extends beyond passive disruption (Baptista et al., 2018). A central mechanism is the amplification of ROS generation, driven both by the redox-active nanoparticle core and the pro-oxidant or redox-cycling properties of specific phytochemicals. This dual ROS induction results in oxidative damage to DNA, proteins, and membrane components, while simultaneously overwhelming bacterial antioxidant defense systems (Rudramurthy et al., 2016). Positively charged nanoparticles, including Ag-NPs and Cu-NPs, form electrostatic interactions with bacterial membranes, causing wall rupture, leakage of intracellular contents, impaired transport, and energy depletion (Wang et al., 2017). Rice-shaped CuO nanoparticles synthesized using *Caesalpinia bonducella* L. extract effectively disrupted *S. aureus* membranes (Sukumar et al., 2020), while Fe-NPs combined with *Hibiscus rosa-sinensis* L. extract induced ROS generation and altered membrane permeability (Buarki et al., 2022). In parallel, phytochemicals released from the nanocarrier can target critical intracellular pathways, including inhibition of DNA gyrase and topoisomerases, disruption of protein synthesis, and interference with metabolic enzymes involved in energy production (Khameneh et al., 2021). Chitosan-based nanoformulations provide intrinsic antimicrobial activity through membrane disruption and metabolic interference (Zou et al., 2016). Functionalization with cationic or hydrophobic components enhances penetration through negatively charged bacterial membranes (Díaz-Puertas et al., 2023; Bekmukhametova et al., 2020). Liposomal formulations of carvacrol and thymol increased membrane disruption, producing inhibition zones of 16 mm and 15.7 mm compared with 15.0 mm and 13.2 mm for free compounds (Liolios et al., 2009).

Importantly, phyto-NPs also target bacterial resistance mechanisms, including efflux pumps and biofilm formation, which act as protective barriers that enhance antibiotic resistance and immune evasion (Kungwani et al., 2024). Nanoparticle penetration into the extracellular polymeric substance (EPS) matrix is facilitated by their size and surface properties, while phytochemicals disrupt quorum sensing (QS), inhibit EPS synthesis, and destabilize established biofilm architecture (Zhao et al., 2020). Liposomal resveratrol demonstrated potent anti-adherence and antibiofilm activity against resistant *S. aureus* strains (Prevete et al., 2023). Efflux-pump inhibition is another critical mechanism, in which liposomal piperine reduced the MIC against MRSA to 3.125 mg/L compared with 100 mg/L for free piperine (Khameneh et al., 2015; Seukep et al., 2020). Xylitol, which is normally unable to penetrate biofilms and is degraded by β-lactamases, exhibited enhanced antibiofilm activity when delivered *via* PLGA nanoparticles (Arunachalam et al., 2023).

A particularly important and mechanistically distinct aspect of phyto-NP activity is their ability to interfere with QS, a central regulatory system governing virulence, toxin production, and biofilm maturation in *S*. *aureus*. In this organism, the accessory gene regulator (Agr) system coordinates population-density-dependent expression of key pathogenic determinants *via* autoinducing peptides (LaSarre and Federle, 2013). Phyto-NPs may disrupt this signaling axis at multiple levels. Surface-associated and released phytochemicals, especially phenolics and flavonoids, have been shown to inhibit AIP synthesis, block ligand-receptor interactions at AgrC, and suppress downstream activation of the AgrA response regulator, thereby attenuating transcription of RNAIII and associated virulence genes (Jakobsen et al., 2017). In addition to direct receptor-level interference, phyto-NPs can indirectly impair QS through membrane perturbation and oxidative stress.

**FIGURE 7:**
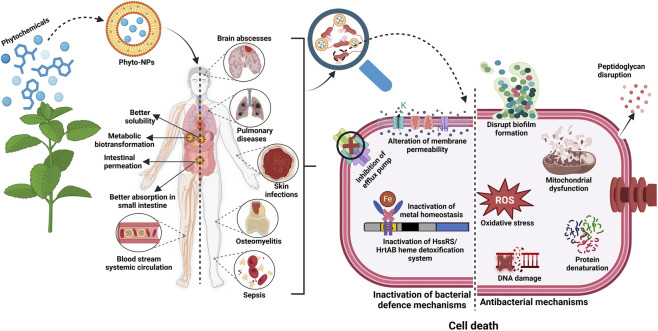
Schematic representation of the antibacterial activity of phytochemical-based nanoparticles against MDR-SA.

Disruption of membrane integrity alters the localization and functionality of membrane-bound QS components, while ROS overproduction can damage regulatory proteins and signaling peptides, further dampening QS signal propagation (Subramaniyan et al., 2021). Nanoparticle-mediated delivery also enhances the local concentration and stability of QS-inhibitory phytochemicals within biofilms, overcoming diffusion limitations that typically reduce the efficacy of free compounds. This is particularly relevant in mature biofilms, where QS gradients are spatially organized and difficult to disrupt (Lu et al., 2019). Importantly, QS inhibition by phyto-NPs does not necessarily exert strong bactericidal pressure, but instead reduces virulence and biofilm robustness, thereby sensitizing bacterial populations to host immune responses and conventional antibiotics. This antivirulence strategy reduces the selective pressure for resistance development while complementing the nanoplatform’s direct bactericidal mechanisms. Figure 7 presents a schematic representation of the antibacterial mechanisms of phyto-NPs against MDR-SA, highlighting their ability to enhance phytochemical efficacy by improving solubility, stability, circulation time, and targeted delivery.

### 4.3 Critical assessment of evidence and methodological limitations

Although many phyto-NPs have demonstrated promising antibacterial efficacy *in vitro*, it is difficult to draw direct comparisons across studies due to substantial methodological variability. The observed MICs of identical phytochemicals, such as curcumin and quercetin, can vary widely due to factors including nanoparticle type, size, surface charge, loading capacity, release profile, bacterial strain, inoculum concentration, and assay conditions. Additionally, the solvent system, culture medium, and incubation conditions introduce ambiguity and reduce the reproducibility of research outcomes. Furthermore, comparisons between free compounds and their NP-based counterparts are not always conducted under identical conditions. Since free phytochemicals often exhibit poor water solubility and limited dispersibility in aqueous solutions, NP-based formulations may contain additional substances, such as surfactants and stabilizers, which can affect their antibacterial performance. Consequently, the increase in antibacterial efficacy from nanosizing might sometimes be exaggerated if proper controls are not used. Moreover, many reported *in vitro* MIC values may not directly translate into clinically achievable concentrations *in vivo*, underscoring the need for pharmacokinetic and pharmacodynamic validation of these systems.

### 4.4 Synergy and multimodal activity

The multimodal antibacterial activity of phyto-NPs arises from interactions between the nanocarrier and bioactive phytochemicals, enabling them to target multiple bacterial functions simultaneously. Unlike conventional antibiotics that target single pathways, phyto-NPs act on multiple bacterial systems simultaneously, reducing the likelihood of resistance development (Díaz-Puertas et al., 2023). Resistance in *S. aureus* may arise from intrinsic factors, spontaneous mutations, or horizontal gene transfer (Sultan et al., 2018; Sun et al., 2019). Alterations in penicillin-binding proteins reduce antibiotic affinity (Lee et al., 2014). Phyto-NP formulations enhance receptor binding, reduce required dosages, and minimize off-target toxicity (Anand et al., 2022).

The synergistic integration of conventional antibiotics with phyto-NPs represents a particularly promising strategy to restore antibiotic efficacy while reducing total antibiotic dosage and selective pressure. Silver nanoparticles synthesized using *Urtica dioica* L. extract increased inhibition zones from 19 mm to 39 mm when combined with antibiotics (Jyoti et al., 2016). Silver nanoparticles derived from *Zea mays* L. waste enhanced rifampicin and kanamycin activity, increasing inhibition zones across multiple pathogens (Patra and Baek, 2017). However, the defining feature of phyto-NPs lies not merely in combinatorial use but in the intrinsic mechanistic synergy between the nanoparticle core and the phytochemical cargo, which is not recapitulated by nanoparticles or phytochemicals alone. These two modalities are functionally integrated, generating a biofunctionalized system that directly underpins the observed potentiation of conventional antibiotics. Increased membrane permeability and biofilm penetration facilitate antibiotic entry, while phytochemical-mediated inhibition of efflux systems and metabolic adaptation enhances intracellular drug retention and target susceptibility (Baptista et al., 2018). In addition, ROS amplification and disruption of the proton motive force further compromise bacterial defense systems, sensitizing cells to antibiotic-induced damage (Hemeg, 2017). Crucially, the nanocarrier enables co-localized and sustained delivery, which may further improve therapeutic selectivity while reducing systemic exposure (Baptista et al., 2018). For example, hyaluronic-acid-coated cinnamaldehyde nanoparticles exploit bacterial hyaluronidase activity for site-specific release against MRSA (Sun et al., 2018). It is also necessary to differentiate between true synergy and additive/parallel activity based on standard pharmacological parameters (Tamma et al., 2012). The fractional inhibitory concentration index (FICI), typically calculated by checkerboard microdilution, remains one of the most reliable approaches for assessing antimicrobial synergy. Typically, FICI scores ≤0.5 indicate synergistic interactions, 0.5 to one denote additive effects, one to four imply indifference, and >4 signify antagonism (Odds, 2003). Nevertheless, despite numerous claims of synergistic activity in phyto-NP research, surprisingly few studies use reliable quantitative assessments of synergism, and most conclusions are based solely on reduced MIC values or expanded inhibition zones. Nevertheless, the current evidence base remains heterogeneous, and direct comparison across studies is complicated by variability in nanoparticle composition, phytochemical loading, bacterial strains, and susceptibility-testing methodologies.

Overall, the synergistic integration of physicochemical disruption and biochemical targeting distinguishes phyto-NPs from their individual components and provides a mechanistic basis for multimodal potentiation of antibiotic activity against MDR-SA. Table 2 details the antibacterial activities of phytochemical-based nanoparticles against MDR-SA, highlighting mechanisms of action and MIC values.

**TABLE 2:** Effects of nanoparticles loaded with phytochemicals against MDR-SA.

Phytochemical-based NPs	Mechanism of action	MIC value	Bacterial species	References
*Tribulus terrestris-*AgNPs	Inhibition of bacterial growth and membrane disruption	Not determined	MDR *S. aureus*	Gopinath et al. (2012)
*Artemisia haussknechtii-*Ag-Cu-TiO_2_NPs	Inhibition of bacterial growth	40mg/L	MDR *S. aureus*	Alavi and Karimi (2018)
*Punica granatum* extract*-*Au-NPs	Inhibition of bacterial growth	15.6mg/L	MRSA	Hussein et al. (2021)
Cinnamon essential oil*-*liposome	Inhibition of biofilm formation	0.25 mg/mL	MRSA	Cui et al. (2016)
Red propolis-polymeric NPs	Inhibition of biofilm formation	100–125 mg/L	MRSA	De Mélo Silva et al. (2020)
Rutin and benzamide-polymeric NPs	Inhibition of biofilm formation and disruption of cell membrane	420 mg/L and 250 mg/L	MDR *S. aureus*	Deepika et al. (2020)
*Capsicum annuum-*Ag-NPs	Inhibition of biofilm formation, generation of superoxide radicals and ROS	80 mg/L	MSSA, MRSA	Ahmed et al. (2018)
Quercetin-Ag-NPs	Inhibition of biofilm formation, generation of superoxide radicals and ROS in MRSA	50 mg/L	MSSA, MRSA	Sadeghi et al. (2024)
Berberine-Au-NPs	Disruption of cell membrane integrity, reduction of viability, ROS production, biofilm inhibition	27.37 mg/L for *S. aureus*, 109.5 mg/L for MRSA	*S. aureus*, MRSA	Sinsinwar and Vadivel (2021)
*Anacardium occidentale*-Ag-NPs	Membrane disruption, ROS and malondialdehyde generation, nucleotide leakage, biofilm inhibition	7.81–31.25 mg/L	*S. aureus,* MSSA, MRSA	Jamil et al. (2016)
Rutin-carbon quantum dots	Cell membrane destruction and increased membrane permeability	32 mg/L	MRSA	He et al. (2019)
*Elettaria cardamomum* essential oil*-*chitosan NPs	Inhibition of bacterial growth	10% *v/v*	MRSA	Jamil et al. (2016)
*Epigallocatechin gallate*-liposome	Inhibition of bacterial growth	16 mg/L	MRSA	Gharib et al. (2013)
*Cotyledon orbiculate*-Ag-NPs	Inhibition of bacterial growth	40 mg/L	MRSA	Díaz-Puertas et al. (2023)
Oleic acid-liposome	Inhibition of bacterial growth	Not determined	MRSA	Huang et al. (2011)
*Aloe vera*-Te-NPs	Inhibition of bacterial growth	11.61 mg/L	MRSA	Medina-Cruz et al. (2021)
*Silybum marianum*-liposome	Inhibition of bacterial growth	125 mg/L	MRSA	Faezizadeh et al. (2015)
*Periploca hydaspidis*-Ag-NPs	Inhibition of bacterial growth	10–20 mg/L	MRSA	Ali et al. (2021)
*Syzygium cumini*-Fe-NPs	Inhibition of bacterial growth	11 mg/L	MRSA	Asghar et al. (2020)
*Stenocereus queretaroensis*-Ag-NPs	Inhibition of bacterial growth	0.313 mg/L	MRSA	Padilla-Camberos et al. (2021)

* NPs: Nanoparticles, Au-NPs: Gold nanoparticles, Ag-NPs: Silver nanoparticles, Fe-NPs: Iron nanoparticles, Te-NPs: Tellurium nanoparticles, TiO_2_-NPs: Titanium dioxide nanoparticles, MDR: Multi-drug resistant, MRSA: Methicillin-resistant *S. aureus,* MSSA: Methicillin-sensitive *S. aureus*.

## 5 Challenges and future directions

Antibiotic resistance continues to pose a major challenge to modern healthcare, with *S*. *aureus* bacteraemia representing a particularly severe clinical problem. Despite substantial efforts in antimicrobial development, the pace of resistance evolution continues to outstrip the introduction of new antibiotics, underscoring the urgent need for alternative and complementary therapeutic strategies (Sultan et al., 2018; Vestergaard et al., 2019).

Phytochemicals and plant-derived nanomaterials have emerged as promising adjuvants and alternatives to conventional antibiotics. Rather than acting solely as physical antimicrobial agents, phyto-NPs function as hybrid systems in which nanoscale delivery enhances the stability, localization, and controlled release of phytochemicals, while the phytochemicals themselves introduce specific biochemical targeting. This convergence of physicochemical and molecular mechanisms underpins their superior efficacy against MDR-SA (Anand et al., 2022; Prevete et al., 2023; Lang et al., 2024).

Despite these advances, substantial challenges remain before phyto-NPs can transition from promising laboratory systems to clinically viable therapies. One major obstacle is the inherent variability of plant extracts, which depends on botanical source, geographical origin, cultivation conditions, and extraction protocols, leading to inconsistent phytochemical composition and biological activity (Patil et al., 2023; Guedes et al., 2024). This variability complicates standardization, reproducibility, and regulatory approval. A major limitation in the current research landscape of phyto-NPs is the poor reproducibility and comparability of antimicrobial efficacy data. Reported MIC values for common phytochemicals vary widely across studies, primarily due to non-standardized experimental parameters. In addition, the physicochemical behavior of phytochemicals, such as low aqueous solubility, instability, and aggregation, can significantly influence apparent activity, leading to inconsistent potency assessments. The absence of standardized protocols and reporting frameworks limits cross-study validation and obscures structure-activity relationships.

Equally important is the limited pharmacological relevance of many *in vitro* findings. Antimicrobial effects are often reported at concentrations that may not be achievable *in vivo* due to constraints related to absorption, metabolic instability, and systemic clearance. While nanoformulation is proposed to enhance bioavailability and tissue distribution, few studies integrate pharmacokinetic-pharmacodynamic considerations or assess whether effective concentrations can be attained and sustained at infection sites, particularly in biofilm-associated infections. This gap restricts the translational interpretation of *in vitro* efficacy. In addition, safety, long-term toxicity, biodistribution, and immunogenicity of complex nanomaterials require far more comprehensive evaluation through well-designed *in vivo* and preclinical studies (Anand et al., 2022; Hulme, 2022).

Another critical limitation in current research is the lack of uniform criteria for assessing antibacterial efficacy and mechanisms of action of nanoparticles. Nanomaterials may exert bactericidal effects through overlapping pathways, including ROS generation, membrane disruption, efflux pump inhibition, and biofilm degradation, making cross-study comparisons difficult (Rudramurthy et al., 2016; Modi et al., 2023). While oxidative stress is widely recognized as a central antibacterial mechanism, the broader impacts of nanoparticles on bacterial metabolism, gene regulation, protein expression, and resistance pathways remain insufficiently characterized (Sun et al., 2019; Zhao et al., 2020).

Further harmonized experimental protocols are required to address these limitations, including standardized susceptibility testing conditions and reporting criteria (Wiegand et al., 2008). Future studies should incorporate pharmacokinetic modeling, tissue distribution analysis, and clinically relevant dosing strategies to bridge the gap between *in vitro* activity and *in vivo* applicability. Rigorous comparative designs using equivalent molar concentrations, controlled formulation variables, and validated synergy metrics are essential to accurately define the contribution of nanoformulation. The integration of advanced models such as biofilm systems, organotypic cultures, and *in vivo* infection models will provide more realistic assessments of therapeutic potential. Such systematic and quantitatively grounded approaches are critical for establishing the true clinical value of phyto-NPs and enabling their rational translation into antimicrobial therapies.

Future progress in this field will require integrating advanced analytical and systems-level approaches. Omics-based strategies, including transcriptomics, proteomics, metabolomics, and genome-wide association studies, offer powerful tools to elucidate nanoparticle-bacteria interactions, adaptive stress responses, and resistance modulation at the molecular level (Vestergaard et al., 2019; Douglas, 2022). Such approaches should be prioritized to identify predictive biomarkers of efficacy, optimize nanoparticle design, and guide rational combination therapies. However, their successful clinical translation depends on rigorous mechanistic elucidation, standardization of phytochemical composition, comprehensive safety and toxicity validation, and the establishment of robust translational research frameworks (Anand et al., 2022; Díaz-Puertas et al., 2023; Guedes et al., 2024). Addressing these challenges through integrated nanotechnology, microbiology, and systems-level approaches will be essential to realize the full potential of phyto-nanotechnologies in the post-antibiotic era (Douglas, 2022; Modi et al., 2023).

## 6 Conclusion

This review highlights the continuing challenge posed by MDR-SA and the growing limitations of conventional antimicrobial strategies. Evidence across phytochemistry and nanomedicine demonstrates that phytochemicals and phytochemical-based nanomaterials offer a credible and conceptually advanced direction for next-generation antibacterial development by integrating natural bioactivity with nanoscale delivery advantages. Their ability to enhance membrane interaction, disrupt biofilms, modulate bacterial defense mechanisms, and support synergistic antibiotic activity positions these systems as integral components of emerging therapeutic frameworks. Nanotechnology strengthens this potential by improving solubility, stability, and site-specific delivery, enabling phytochemicals to reach complex infection niches with greater precision and potency. Rather than relying on escalating systemic antibiotic exposure, rationally engineered nanosystems provide multimodal, mechanism-driven intervention capable of reshaping host-pathogen interactions. Phyto-nanomaterials further reduce the likelihood of resistance development by simultaneously targeting multiple bacterial pathways, enabling lower drug dosages and improved therapeutic durability. Despite these advances, successful translation requires deeper mechanistic resolution, consistent evaluation criteria, and comprehensive safety and toxicity assessment. Variability in plant-derived extracts, limited standardization, and incomplete understanding of nanoparticle-bacteria interactions continue to constrain clinical progress. Future work must prioritize rigorous *in vivo* validation, improved reproducibility, and integration of systems-level analytical approaches to clarify antibacterial mechanisms and guide rational design. Phytochemical-based nanosystems represent a forward-looking, mechanism-centered strategy for managing MDR-SA infections. Their continued development will depend on sustained interdisciplinary research that can transform experimental promise into clinically reliable tools for the post-antibiotic era.
